# SERPING1 Reduces Cell Migration via ERK‐MMP2‐MMP‐9 Cascade in Sorafenib‐ Resistant Hepatocellular Carcinoma

**DOI:** 10.1002/tox.24434

**Published:** 2024-10-30

**Authors:** Ching‐Chuan Hsieh, Yuh‐Harn Wu, Yi‐Li Chen, Chun‐I Wang, Chao‐Jen Li, I‐Hsiu Liu, Chen‐Wei Chou, Yang‐Hsiang Lin, Po‐Shuan Huang, Te‐Chia Huang, Cheng‐Yi Chen

**Affiliations:** ^1^ Division of General Surgery, Chang Gung Memorial Hospital Chiayi Taiwan; ^2^ Department of Cell Biology and Anatomy, College of Medicine National Cheng Kung University Tainan Taiwan; ^3^ Department of Biochemistry, School of Medicine China Medical University Taichung Taiwan; ^4^ Department of General & Gastroenterological Surgery, An Nan Hospital China Medical University Tainan Taiwan; ^5^ Liver Research Center, Chang Gung Memorial Hospital Taoyuan Taiwan; ^6^ Department of Biochemistry, College of Medicine Chang Gung University Taoyuan Taiwan; ^7^ Graduate Institute of Biochemical and Biomedical Engineering Chang Gung University Taoyuan Taiwan

**Keywords:** cancer progression, drug resistance, hepatocellular carcinoma, SERPING1, sorafenib

## Abstract

Hepatocellular carcinoma (HCC) is the most common primary hepatic malignant tumor, and it ranks 2nd in terms of mortality rate among all malignancies in Taiwan. Sorafenib is a multiple tyrosine kinase inhibitor that suppresses tumor cell proliferation and angiogenesis around tumors via different pathways. However, the survival outcome of advanced HCC patients treated with sorafenib is still unsatisfactory. Unfortunately, there are no clinically applicable biomarkers to predict sorafenib therapeutic efficiency in HCC thus far. We found that serpin peptidase inhibitor, clade G, member 1 (SERPING1) is highly associated with overall and recurrence‐free survival rates in HCC patients and is also highly correlated with several clinical parameters. SERPING1 expression was increased with sorafenib in both the HCC cell extract and conditioned medium, which was also observed in sorafenib‐resistant HepG2 and Huh7 cells. Sorafenib decreased cell viability and migration, which was similar to the effect of SERPING1 in HCC progression. Moreover, sorafenib inhibited both MMP‐2 and MMP‐9 activity and enhanced the expression of p‐ERK in HCC cells. In summary, sorafenib reduces HCC cancer progression might through the p‐ERK‐MMP‐2‐MMP‐9 cascade via upregulation of SERPING1. In the present study, the roles and molecular mechanisms of SERPING1 and its value as a marker for predicting sorafenib resistance and progression in HCC patients were examined. The results of the present study provide a deep understanding of the roles of SERPING1 in HCC sorafenib resistance, which can be applied to develop early diagnosis and prognosis evaluation methods and establish novel therapeutic targets for specifically treating HCC.

## 1 Introduction

Hepatocellular carcinoma (HCC) stands as the most prevalent primary hepatic malignant tumor and is the second leading cause of cancer‐related mortality globally [1]. The majority of HCC diagnoses correlate with underlying chronic liver diseases, notably chronic hepatitis B and hepatitis C infections, often presenting with nonspecific symptoms in the early stages. Alarmingly, roughly 70% of patients receive an HCC diagnosis with limited eligibility for curative therapy. Sorafenib, a multikinase inhibitor, exerts its effects by suppressing tumor cell proliferation and angiogenesis via diverse pathways [2].

Sorafenib has demonstrated the potential to extend the median survival time of HCC patients by approximately 3–5 months [2]. However, various mechanisms and pathways contributing to acquired resistance to sorafenib have been identified, including the epithelial‐mesenchymal transition (EMT) mechanism, activation of hypoxia‐inducible pathways, the phosphatidylinositol‐3‐kinase (PI3K)/Akt pathway, and Janus kinase‐signal transducer and activator of transcription (JAK–STAT) pathways [2]. Regrettably, the lack of reliable markers for predicting sorafenib efficacy in HCC remains a significant challenge. Consequently, there is an imperative need to elucidate the dysregulated mechanisms and molecules driving resistance to sorafenib in the progression of HCC.

SERPING1 is a protease inhibitor belonging to the serpin superfamily [3]. The SERPING1 gene, located on chromosome 11, encodes a glycosylated plasma protein named C1‐inhibitor, which can prohibit the activation of the classical complement pathway [4]. SERPING1 is an independent predictor of survival in lung squamous cell carcinoma patients and may be defined as a prognostic marker [5]. In addition, levels of SERPING1 can provide a reference for low‐risk prostate cancer patients to accept active surveillance and reduce overtreatment [6]. Therefore, SERPING1 may play an important role in prostate cancer and can serve as a novel marker in prostate cancer diagnosis and prognosis prediction. Previously, Fornvik et al. reported that SERPING1 is upregulated in human glioblastoma (astrocytoma grade IV) at both the mRNA and protein levels [7]. There is growing evidence of the presence of complement components and several regulatory proteins in most types of tumor cells [8].

Despite its significance, the precise mechanisms through which SERPING1 influences sorafenib resistance and HCC progression remain elusive. Our study sheds light on this aspect by revealing a strong correlation between SERPING1 expression and overall and recurrence‐free survival rates in HCC patients. Additionally, SERPING1 exhibits high correlations with various clinical parameters, including alpha‐fetoprotein (AFP) level, predicted metastasis risk signature score, primary tumor size, and pathological stage. Notably, SERPING1 is upregulated in both sorafenib‐stimulated parental and sorafenib‐resistant (SR) HCC cells. Remarkably, SERPING1 overexpression significantly attenuates cell migration, mirroring the effects observed with sorafenib treatment in HCC cells. Moreover, sorafenib‐induced suppression of HCC progression appears to be mediated, at least in part, by upregulating SERPING1 expression and activating the p‐ERK‐MMP‐2‐MMP‐9 cascade. Hence, our findings suggest a pivotal role for SERPING1 dysregulation in HCC, contributing to sorafenib resistance and cancer development. Furthermore, SERPING1 emerges as a promising candidate marker for HCC. Understanding the intricate molecular and cellular mechanisms underlying SERPING1‐mediated sorafenib resistance could unveil new avenues for complementary therapies in hepatoma treatment.

## 2 Materials and Methods

### 2.1 Cell Culture and Treatments

The human hepatoma cell lines HepG2, J7, and Huh7 were cultured routinely at 37°C in a humidified atmosphere comprising 95% air and 5% CO_2_ in Dulbecco's Modified Eagle's Medium (DMEM; Invitrogen, Grand Island, NY) supplemented with 10% fetal bovine serum (HyClone, Road Logan, UT). Notably, HepG2 and Huh7 cells represent well‐differentiated HCC cells [9]. Previous studies have utilized Huh7 cells to investigate cancer cell motility [10]. Thus, these cells were challenged with sorafenib (Sigma‐Aldrich) and SERPING1 recombinant protein (PeproTech, Rehovot, Israel).

### 2.2 MTT Assay

Cell proliferation ability was assessed using the (3‐(4,5‐dimethylthiazol‐2‐yl)‐2,5‐diphenyltetrazolium bromide) (MTT) assay (Sigma‐Aldrich). Initially, cells (5 × 10^3) were seeded onto 96‐well plates and allowed to adhere overnight. Following the completion of treatment, 20 μL of MTT solution was added to each well and incubated for 3 h at 37°C. Subsequently, the absorbance at 490 nm was quantified using a SpectraMax microplate reader (Molecular Devices, San Jose, CA).

### 2.3 Preparation of Conditioned Medium

The conditioned medium was collected and centrifuged at 2000 × *g* for 5 min to eliminate intact cells, followed by concentration using spinning columns with a 3 kDa molecular weight cutoff (Amicon Ultra, Millipore); the samples were stored at −80°C for subsequent experiments.

### 2.4 Western Blotting

Total cell extracts (10 μg of protein) were resolved by 10% SDS–PAGE, transferred onto an Immobilon polyvinylidene difluoride membrane (Amersham Biosciences), and immunoblotted with specific primary antibodies against SERPING1 (GeneTex), p‐ERK (ABclonal, Wobum, MA, USA), ERK (ABclonal), and β‐actin (GeneTex) overnight at 4°C. Following primary antibody incubation, the membrane was incubated with appropriate HRP‐conjugated secondary antibodies for 1 h at room temperature. Finally, immunoreactive bands were visualized using the chemiluminescence method with an ECL detection kit (Millipore).

### 2.5 Wound Healing Assay

The wound healing scratch assay was employed to assess cell migration. Confluent cells were cultured in DMEM (Invitrogen, Grand Island, NY) and treated with various doses (0–2 μg/mL) of SERPING1. After 24 h, the impact of SERPING1 on wound closure was evaluated. Images of the wounded cell monolayers were captured at 0, 12, 16, and 24 h post‐wounding using a microscope (model IX‐70; Olympus, Tokyo, Japan). The wound healing rate was quantified using ImageJ software (Universal Imaging Co., Ltd., UK).

### 2.6 Establishment of Sorafenib‐Resistant HCC Cells

Hepatoma cells, such as HepG2 or Huh7 cells, were cultured in medium supplemented with incrementally escalating concentrations of sorafenib ranging from 0.5 to 7 μM for a duration of 6 months, following established protocols [11]. Subsequently, after successful establishment, HepG2 SR (HepG2‐SR) and Huh7 SR (Huh7‐SR) cells were maintained in medium containing 7 μM sorafenib.

### 2.7 In Vitro Migration Assay

Hepatoma cells (10^4^ cells/well) were seeded onto the upper chambers of non‐Matrigel‐coated Transwell plates (Becton‐Dickinson) with serum‐free medium. The medium in the lower chamber was supplemented with 10% FBS. Following a 24‐h incubation period at 37°C, cells that migrated through the filter were enumerated.

### 2.8 Immunohistochemistry

The utilization of archived formalin‐fixed, paraffin‐embedded tissue blocks was authorized by the Institutional Review Board of National Cheng Kung University Hospital. Tissue slides obtained from HCC patients underwent evaluation via immunohistochemistry and hematoxylin/eosin staining, employing antibodies against SERPING1 (GeneTex) and following the avidin‐biotin complex method, as previously outlined [12]. Immunoreactivity for SERPING1 was visualized using DAB/nickel substrate (Vector Laboratories, Burlingame, CA).

### 2.9 Statistical Analysis

All values are presented as means ± standard deviations (SDs). Experimental differences among groups were assessed using two‐way ANOVA, Student's *t*‐test, chi‐square test, or Fisher's exact test, as appropriate. The Kaplan‐Meier method was employed to analyze the overall survival of test mice, and prognostic significance was determined using the log‐rank test. Statistical significance was set at a *p* value of < 0.05.

## 3 Results

HCC treatment continues to pose challenges, with targeted therapeutic agents showing limited efficacy and tumors frequently developing acquired resistance following exposure [13]. To address this, we examined sorafenib resistance gene expression profiles from the Gene Expression Omnibus database (GSE94550) and the Roessler Liver microarray dataset, integrated with clinical information from the Oncomine database [14], to identify potential candidates associated with HCC sorafenib resistance in clinical samples. Therefore, we analyzed two microarray datasets and intersected them to identify promising targets. Specifically, in the Roessler Liver microarray dataset, candidates were filtered based on a > 1.1‐fold change in expression levels between patients in the bottom 25% and top 25% in terms of survival time. Subsequently, genes identified in the Roessler Liver dataset were overlapped with sorafenib‐regulated genes (those altered > 1.5‐fold with vs. without sorafenib treatment), as reported by Kelly Regan et al. From this analysis, three sorafenib resistance‐related genes aberrantly expressed in HCC specimens were selected for further investigation.

**FIGURE 1:**
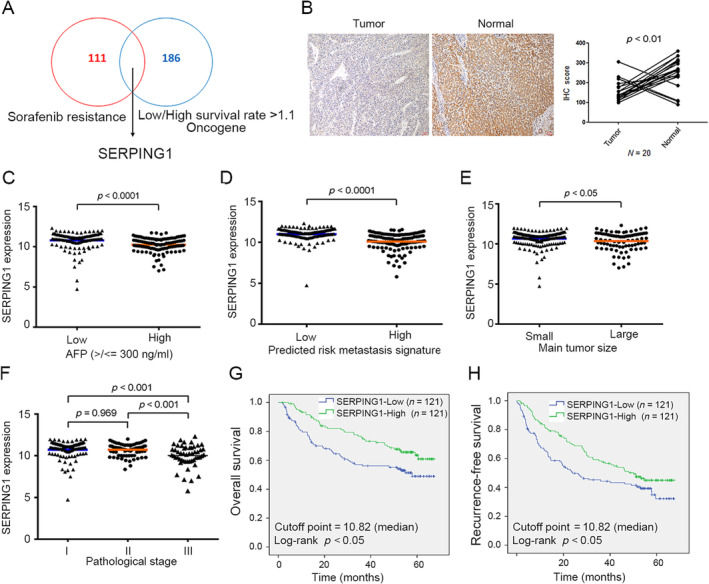
High expression of SERPING1 indicates better patient survival. (A) The two datasets, the Roessler Liver microarray and Huh7 sorafenib resistance array datasets, were analyzed and intersected to find potential candidates. SERPING1 emerged as a potential candidate and was selected for further study. (B) Immunohistochemistry staining revealed that SERPING1 expression was lower in clinical HCC tissues compared to normal tissues. The immunohistochemistry staining scores were quantified (C‐F). SERPING1 levels were significantly lower in patients with high alpha‐fetoprotein (AFP) levels, high predicted metastasis risk signature score, large tumor sizes, and advanced pathological stages. (G, H) Kaplan‐Meier survival curves of two groups of patients defined by different SERPING1 expression levels were established on the basis of Roessler liver microarray scores (*n* for each group = 121, cutoff point = 10.82 (the median), log‐rank *p* < 0.05). Patients with high SERPING1 levels have better survival than those with low levels.

These genes were verified and narrowed down based on the clinical significance of the 5‐year survival rate or other clinical parameters (Figure 1A). Among the candidate genes, we determined which genes to study by literature review. SERPING1 is a protease inhibitor belonging to the serpin superfamily [3]. SERPING1 was upregulated by sorafenib and selected for further study, as it is an independent predictor of survival in lung cancer patients and may be defined as a prognostic marker [5]. Firstly, we examined the levels of SERPING1 in HCC clinical specimens. We evaluated SERPING1 expression in 20 HCC specimens using immunohistochemistry. Among these, 16 tumor tissues exhibited lower SERPING1 expression compared to corresponding normal tissues (Figure 1B, left). Quantitative analysis of IHC staining scores revealed that SERPING1 expression was significantly downregulated in tumor tissues compared to normal controls (Figure 1B, right). SERPING1 might play a tumor suppressor role in HCC.

The Roessler liver microarray dataset provides several types of clinical information, including pathological stage, tumor‐node‐metastasis (TNM) stage, AFP, alanine aminotransferase (ALT), tumor size, overall survival rate, recurrence status, and predicted metastasis risk signature score. Statistical analysis was conducted to assess the correlation between SERPING1 expression and various clinical parameters, aiming to elucidate the role of sorafenib‐regulated SERPING1 in HCC progression (Tables 1, 2, Table S1). Notably, SERPING1 expression exhibited significant correlations with serum AFP (*p* < 0.001), tumor size (*p* = 0.047), and CLIP score (*p* = 0.002) (Table 1). Univariate analysis revealed that cirrhosis (*p* = 0.009), serum AFP (*p* = 0.011), tumor size (*p* = 0.001), CLIP score (*p* < 0.001), AJCC stage (*p* < 0.001), and high SERPING1 levels (*p* = 0.011) were significant predictors of improved overall survival (Table 2). Multivariate analysis further identified cirrhosis (*p* = 0.035, HR = 4.545, CI = 1.115–18.522), tumor size (*p* = 0.023, HR = 1.635, CI = 1.070–2.497), CLIP stage (*p* = 0.001, HR = 2.175, CI = 1.388–3.409), and high SERPING1 levels (*p* = 0.033, HR = 0.640, CI = 0.425–0.964) as independent factors associated with improved overall survival (Table 2). Moreover, in terms of recurrence‐free survival, univariate analysis identified tumor size (*p* = 0.045), CLIP score (*p* = 0.002), AJCC stage (*p* < 0.001), and high SERPING1 levels (*p* = 0.026) as significant predictors of better outcomes (Table S1).

**TABLE 1:** Association of SERPING1 level (Roessler liver array) with clinicopathologic indicators of hepatocellular carcinoma.

Factors	Group		SERPING1 (mean ± SE)	*p*
Age	< 60 years		10.4878 ± 0.0769	0.104
	≥ 60 years		10.7578 ± 0.1333	
Sex	Male		10.5557 ± 0.0743	0.639
	Female		10.4610 ± 0.1404	
Cirrhosis	Absent		10.5211 ± 0.2910	1.162
	Present		10.5455 ± 0.0688	
Serum AFP	< 300		10.8001 ± 0.0941	< 0.001*
(ng/ml)	≥ 300		10.2460 ± 0.0889	
Tumor size	< 5 cm		10.6450 ± 0.0792	0.047*
	≥ 5 cm		10.3557 ± 0.1209	
CLIP	0–1		10.6447 ± 0.0725	0.002*
	≥ 2		10.1348 ± 0.1581	
AJCC stage	I		10.6640 ± 0.0979	0.094
	≥ II		10.4381 ± 0.0918	

Abbreviations: AFP, alpha‐fetoprotein; AJCC, American Joint Committee on Cancer 2017; CLIP, Cancer of the Liver Italian Program score.

* *p* < 0.05.

**TABLE 2:** Prognostic significance of clinicopathologic indicators and SERPING1 for overall survival in the Roessler liver array.

Factor	OS univariate				OS multivariate		
	Group	HR	95% CI	*p*	HR	95% CI	*p*
Age	< 60/≥ 60 years	0.842	0.504–1.406	0.510			
Sex	Female/Male	1.858	0.901–3.833	0.094			
Cirrhosis	±	5.093	1.255–20.671	0.023*	4.545	1.115–18.522	0.035*
Serum AFP	< 300/≥ 300 ng/mL	1.686	1.126–2.527	0.011*			NS
Tumor size	< 5/≥ 5 cm	1.960	1.309–2.933	0.001*	1.635	1.070–2.497	0.023*
CLIP	0–1/≥ 2	2.811	1.832–4.313	< 0.001*	2.175	1.388–3.409	0.001*
AJCC stage	I/≥ II	2.278	1.483–3.500	< 0.001*			NS
SERPING1	Low/high	0.591	0.394–0.886	0.011*	0.640	0.425–0.964	0.033*

Abbreviations: AFP, alpha‐fetoprotein; AJCC, American Joint Committee on Cancer 2017; CLIP, Cancer of the Liver Italian Program score; OS, overall survival.

* *p* < 0.05.

Consequently, SERPING1 expression was significantly decreased with higher AFP levels, predicted metastasis risk signature (Figure 1C,D), primary tumor size, and more advanced pathological stage of HCC (Figure 1E,F). These clinical parameter correlation data indicate that SERPING1 expression is highly associated with HCC progression and could possibly be selected as a useful prognostic marker for HCC. Moreover, the effect of SERPING1 expression on the survival rate was also examined. It was clear that patients with high expression of SERPING1 had a better overall survival rate (log‐rank *p* < 0.05; SERPING1 High: stand error, 1.998; 95% CI, 48.898–56.731; SERPING1 Low: Stand error, 2.499; 95% CI, 38.114–47.911) and recurrence‐free survival rate (log‐rank *p* < 0.05; SERPING1 High: Stand error, 2.279; 95% CI, 38.922–47.857; SERPING1 Low: Stand error, 2.546; 95% CI, 29.439–39.418) (Figure 1G,H). Based on the evidence, we suggest that SERPING1 might be a tumor suppressor in HCC progression.

**FIGURE 2:**
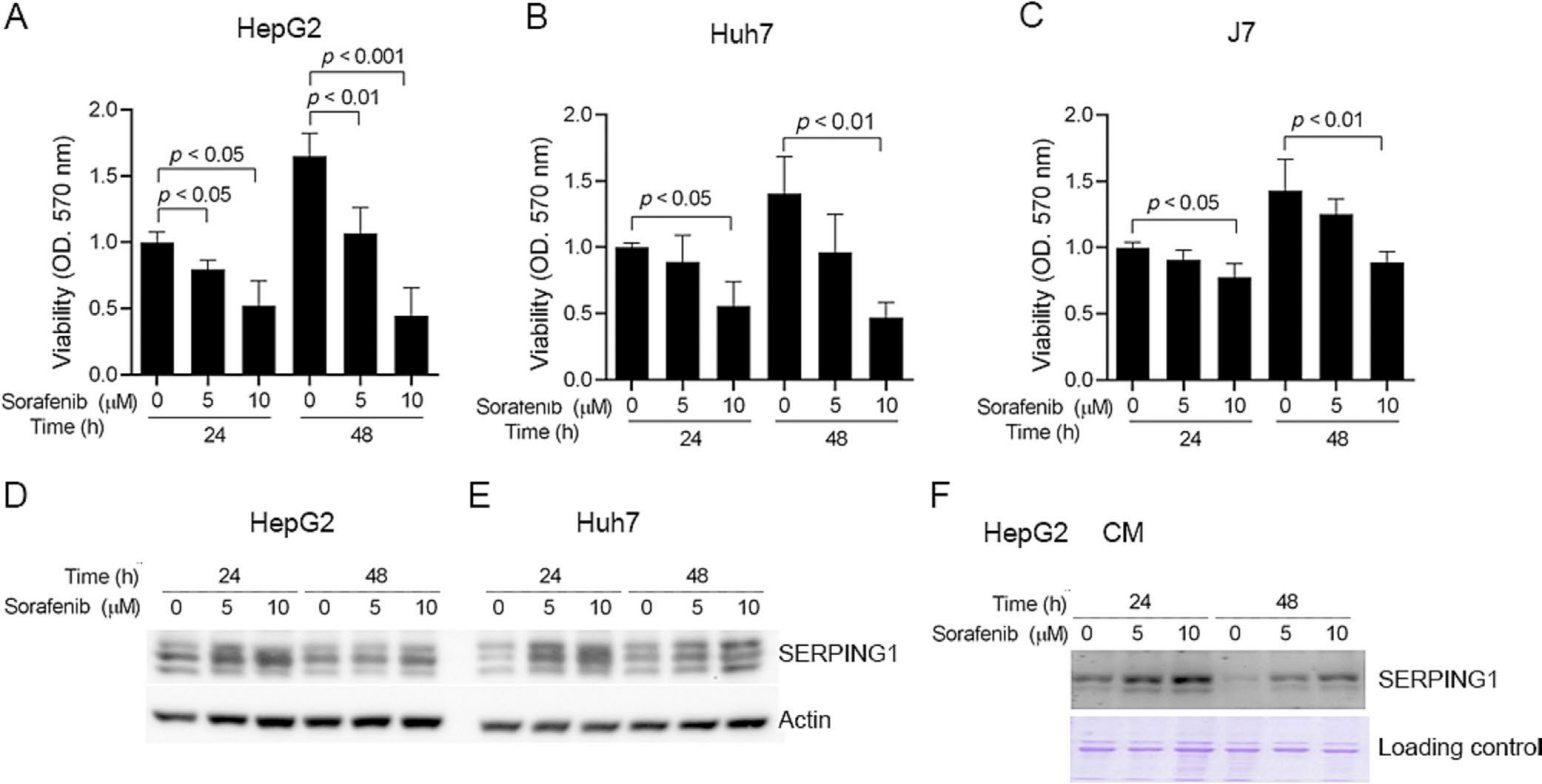
Sorafenib increases SERPING1 expression and cell viability. (A‐C) HCC cell viability was determined by MTT assay at 24 and 48 h after sorafenib (0–10 μM) treatment in HepG2 (A), Huh7 (B), and J7 (C) cells. Sorafenib decreased cell viability in a dose‐ and time‐dependent manner. (D–F) The SERPING1 levels in both the cell lysate (D, E) and conditioned medium (CM) (F) with various doses (5–10 μM) of sorafenib stimulation were determined using Western blotting in HepG2 (D, F) and Huh7 (E) cells. Sorafenib induced SERPING1 expression in both cell lysate and CM in HCC cells. Coomassie Brilliant Blue staining was used as a loading control.

To study the effect of sorafenib on HCC, first, cell viability was determined by MTT assay after 5 and 10 μM sorafenib stimulation. The viability of HepG2, Huh7, and J7 cells was significantly inhibited by sorafenib at 24 and 48 h (Figure 2A–C). The inhibitory effect of sorafenib on viability was time and dose‐dependent. In addition, we assessed whether SERPING1 regulation could be influenced by sorafenib, and the expression of SERPING1 was increased with 5 and 10 μM sorafenib treatment at 24 and 48 h in HepG2 and Huh7 cells according to Western blotting (Figure 2D,E). Moreover, sorafenib‐mediated SERPING1 upregulation was also observed in the conditioned medium of HepG2 cells, suggesting that SERPING1 might be a marker for sorafenib sensitivity in HCC (Figure 2F).

**FIGURE 3:**
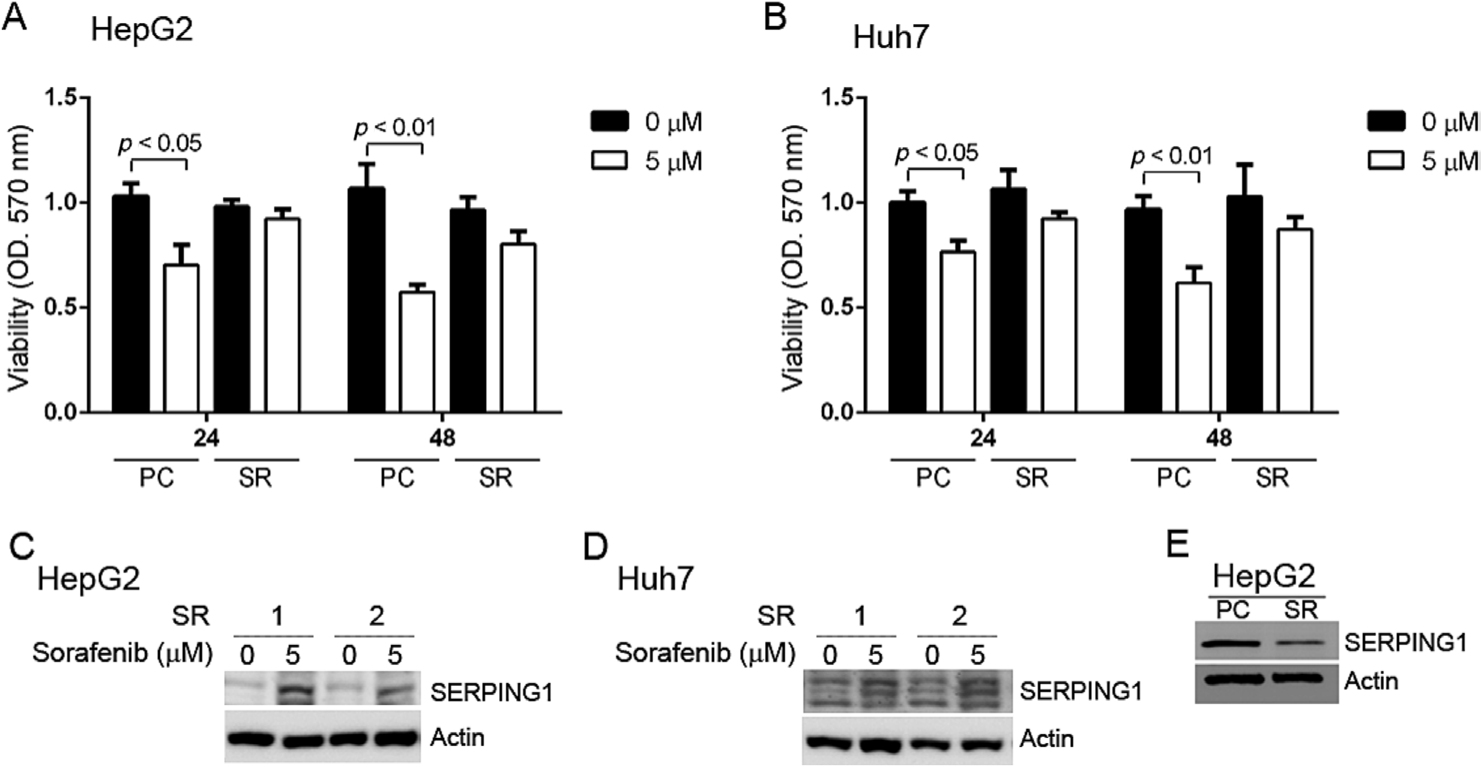
Sorafenib‐resistant cells have a higher viability rate after sorafenib treatment than parental cells. (A, B) Sorafenib‐resistant (SR) cells were established, and the cell viability of SR cells was not significantly influenced by 5 μM sorafenib at either 24 or 48 h. However, the cell viability of parental cells (PCs) was significantly decreased at 24 and 48 h after 5 μM sorafenib treatment in HepG2 (A) and Huh7 (B) cells. (C, D) The SERPING1 level was examined in the cell lysate of sorafenib‐resistant (SR; 1,2: Two resistant cell lines) HCC cells, and SERPING1 expression was induced with 5 μM sorafenib stimulation in HepG2 (C) and Huh7 (D) cells. (E) The SERPING1 levels were determined in HepG2‐PC and HepG2‐SR cells by Western blotting. SERPING1 was lower expressed in SR compared to PC.

Moreover, we established HepG2 and Huh7 SR cells via chronic low‐dose sorafenib stimulation to verify whether the effect could be observed in SR cells (Figure 3). The cell viability of the HepG2 and Huh7 parental cells (PCs) with 5 μM sorafenib stimulation for 24 and 48 h was decreased according to the MTT assay; however, the effect was not observed in HepG2‐SR and Huh7‐SR cells (Figure 3A,B). Based on the results, we suggest that SR cells have more protection against sorafenib toxicity. Therefore, we assessed whether the effect could be observed in SR HepG2 and Huh7 cells with 5 μM sorafenib, and the phenotype was similar to that in HCC PCs stimulated with sorafenib (Figure 3C,D). Additionally, the SERPING1 levels in both HepG2‐PC and ‐SR cells were also determined, and we found that SERPING1 expression was obviously lower in resistant cells (Figure 3E), which meant that SERPING1 might play a tumor suppressor role in cancer progression, which was consistent with the clinical parameter correlation results (Figure 1). Based on these observations, we infer that reduced SERPING1 expression contributes to increased aggressiveness and resistance to sorafenib in HCC. However, the detailed mechanism of sorafenib in both sorafenib‐sensitive and SR cells needs to be elucidated further.

**FIGURE 4:**
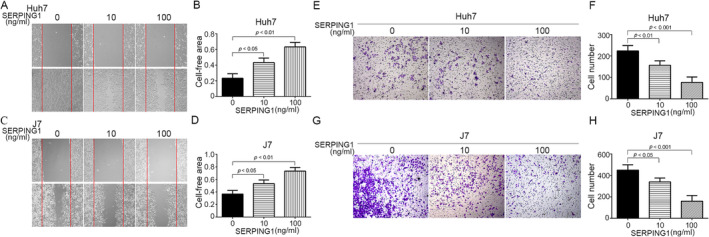
SERPING1 decreases cell migration. (A‐H) Huh7 (A, B, E, F) and J7 (C, D, G, H) cells were stimulated with 10 or 100 ng/mL recombinant SERPING1 protein and subjected to wound healing (A, C) and a Transwell (E, G) analyses with quantification (B, D, F, H). Treatment with recombinant SERPING1 decreased cell migration.

In summary, we found that SERPING1 was highly correlated with survival rates and numerous parameters and was regulated by sorafenib in both parental and resistant cells. Therefore, we investigated which functions of SERPING1 are involved in cancer progression and resistance to sorafenib in HCC. Previously, Fornvik and colleagues demonstrated that the survival rate is increased in rats inoculated intracerebrally with glioma cells precoated with anti‐SERPING1 antibody [7]. Hence, we stimulated HCC cells with various doses of 10 and 100 ng/mL SERPING1 recombinant protein and analyzed wound healing to determine whether SERPING1 can influence cell motility. The wound healing data indicated that SERPING1 decreased Huh7 and J7 cell migration in a dose‐dependent manner (Figure 4A,C), and the results were quantified (Figure 4B,D). Additionally, we utilized a Transwell assay to determine whether SERPING1 can inhibit cell migration. A similar phenomenon was observed: SERPING1 significantly decreased cell migration in Huh7 (Figure 4E,F) and J7 (Figure 4G,H) cells in a dose‐dependent manner (Figure 4E–H).

**FIGURE 5:**
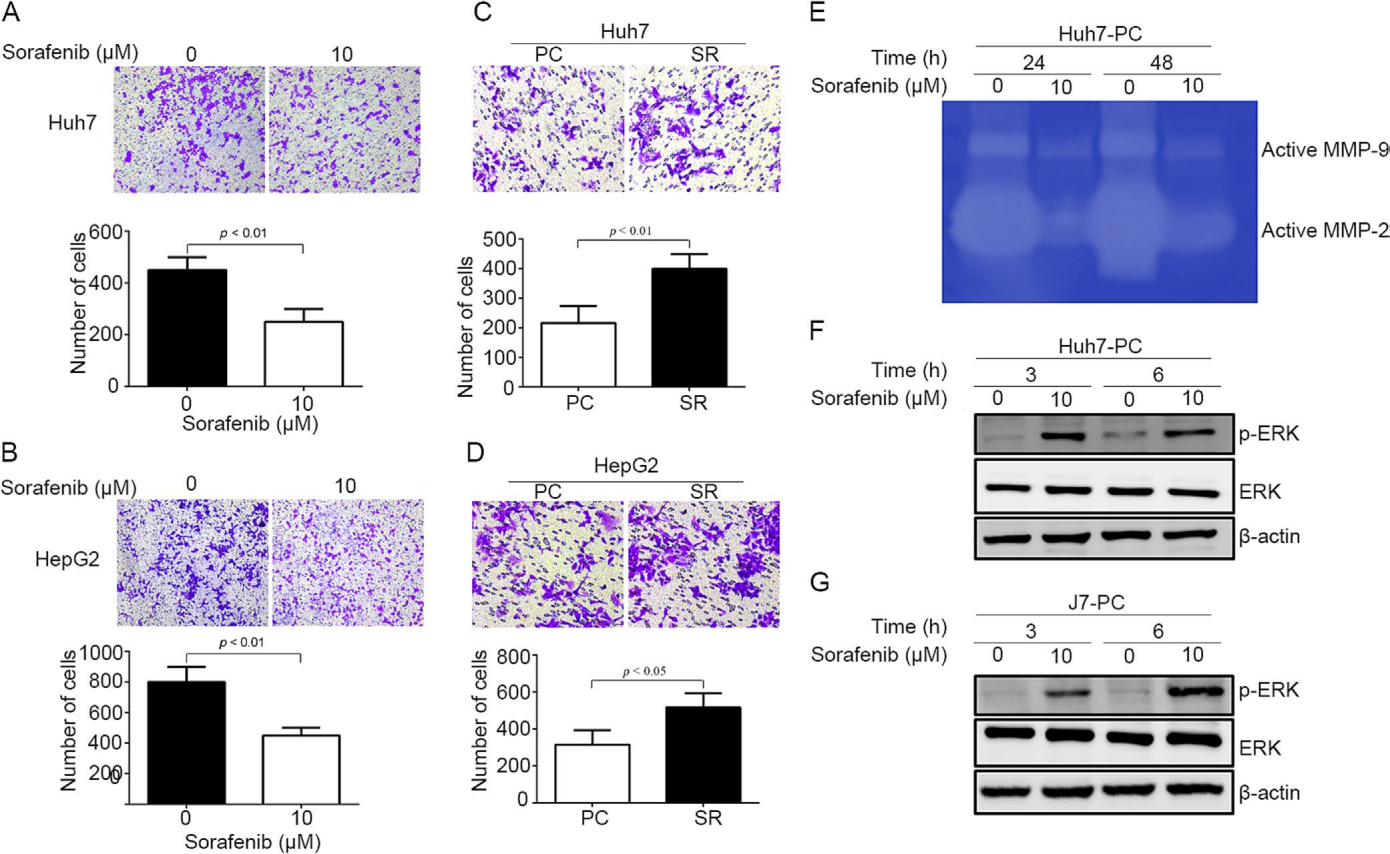
MMP activity and p‐ERK might be involved in sorafenib‐reduced cell migration. (A, B) Sorafenib (10 μM) reduced cell migration using a Transwell assay in both Huh7 (A) and HepG2 (B) cells, whose results were also quantified (lower panel, A, B,). (C, D) Sorafenib‐resistant (SR) cells had higher cell migration compared with parental cells (PC) in both Huh7 (C) and HepG2 (D) cells by a Transwell assay analysis. (E‐G) Sorafenib (10 μM) reduced the activity of active‐form MMP‐2 and MMP‐9 by zymography analysis and enhanced the levels of p‐ERK using Western blotting in Huh7‐PC (E, F) and J7‐PC (G) cells.

The sorafenib effect on cell migration in HCC cells was also examined; sorafenib (10 μM) significantly reduced cell migration in both Huh7 and HepG2 cells (Figure 5A,B). In addition, the Huh7‐SR and HepG2‐SR cells have higher migratory ability compared to Huh7‐PC and HepG2‐PC cells using a Transwell assay (Figure 5C,D). SERPING1, a protease inhibitor belonging to the serpin superfamily, can modulate extracellular matrix metalloproteinases (MMPs) activity, such as MMP‐2 and MMP‐9, to influence cancer cell motility [3]. Hence, we determined whether sorafenib can regulate MMP activity. Zymography data indicated that sorafenib inhibited the activity of active‐form MMP‐2 and MMP‐9 at 24 and 48 h in Huh7‐PC cells (Figure 5E). Furthermore, we analyzed which signaling pathways can be modulated by sorafenib. Western blotting showed that phosphorylation (p)‐ERK was enhanced after 10 μM sorafenib stimulation for 3 and 6 h in both Huh7‐PC and J7‐PC cells (Figure 5F,G).

Furthermore, we exposed HCC cells (Huh7 and J7) to 5 μM sorafenib for 24 h. Interestingly, we observed a decrease in p‐ERK levels following this treatment (data not shown). This finding suggested a complex regulatory effect of sorafenib on p‐ERK. We speculate that short‐term sorafenib exposure may activate p‐ERK as part of a compensatory mechanism, which could support alternative survival pathways such as autophagy in HCC cells. In contrast, prolonged sorafenib treatment appears to induce substantial toxicity, thereby disrupting these compensatory mechanisms and leading to a reduction in p‐ERK levels. Collectively, we suggest that SERPING1 plays a role in SR HCC cells might through modulation of cell migration, which might involve in sorafenib‐regulated p‐ERK‐MMP‐2‐MMP‐9 cascade.

**FIGURE 6:**
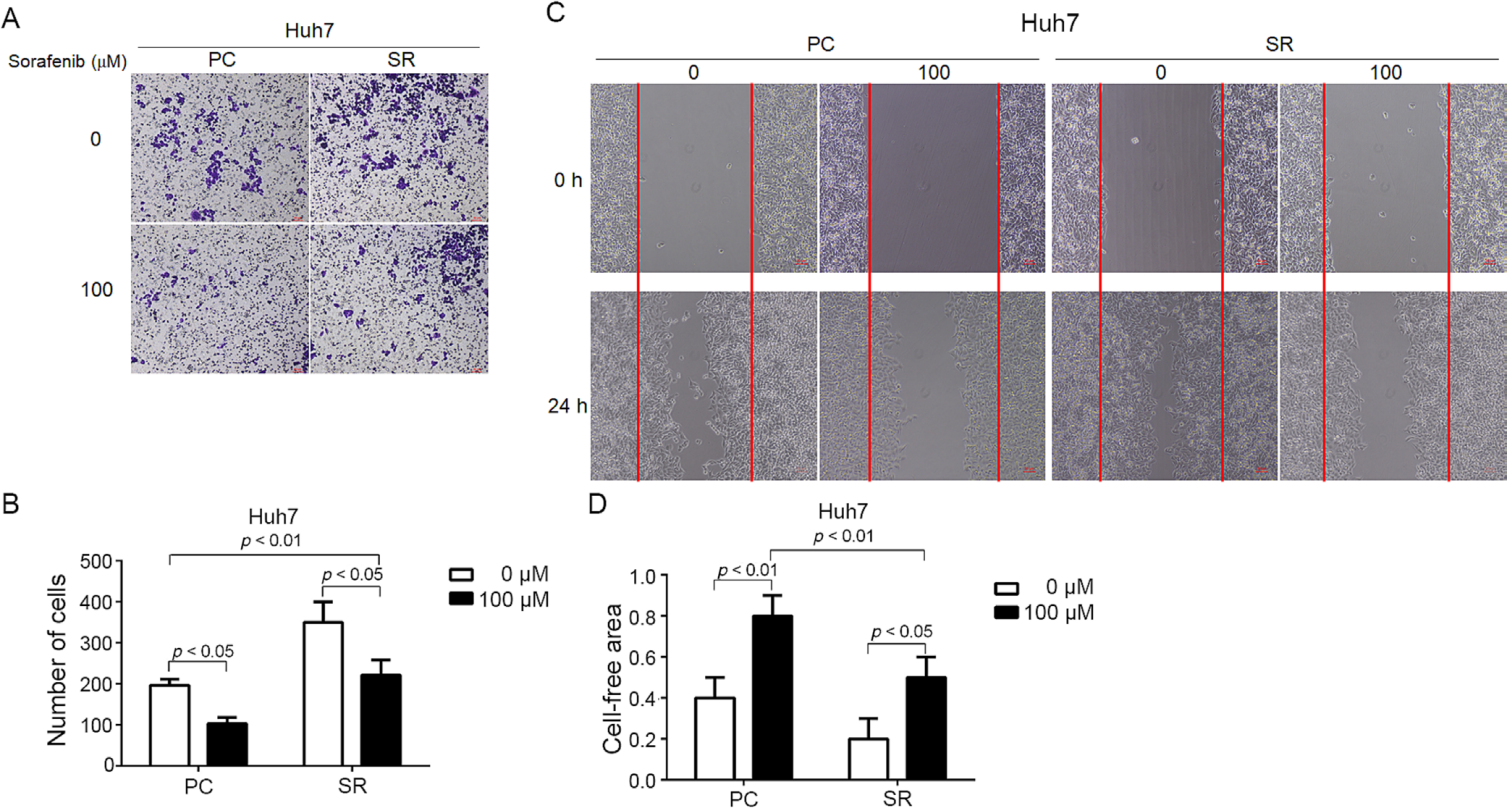
SERPING1 reverses sorafenib‐resistant cell‐induced cell migration. (A‐D) Baseline cell migration ability was higher in SR cells than in PC cells. Treatment with recombinant SERPING1 protein reduced the number of migratory cells in both PC and SR cells, as demonstrated by the Transwell (A) and wound healing (C) assays. Notably, even with SERPING1 stimulation, the number of migratory SR cells remained higher than that of PC cells. The results were also quantified (B, D).

To assess whether SERPING1 influences the migratory phenotype of SR cells, we observed that SERPING1 expression was reduced in SR cells compared to PC cells, as determined by Western blotting (Figure 3E). To evaluate whether SERPING1 affects the migratory phenotype of SR cells, we conducted Transwell migration and wound healing assays. The Transwell assay demonstrated that SR cells had a higher baseline migratory capacity compared to PC cells. However, stimulation with 100 ng/mL recombinant SERPING1 protein resulted in a reduction in the number of migratory cells in both PC and SR cells within the Huh7 line (Figure 6A,B). Despite this decrease, SR cells consistently exhibited significantly higher migration than PC cells, both with and without SERPING1 stimulation (Figure 6A,B). Similar results were observed in the wound healing assay (Figure 6C,D). Collectively, these findings suggest that lower SERPING1 levels in SR cells contribute to enhanced migratory capacity, which can be notably attenuated by SERPING1 stimulation.

## 4 Discussion

We found that high SERPING1 expression was significantly associated with better overall survival and recurrence‐free survival rates, and SERPING1 expression was decreased in late‐stage HCC tissues compared with early‐stage HCC tissues, which indicates that SERPING1 might be a tumor suppressor (Figure 1). HCC cell viability and SERPING1 expression were significantly regulated by sorafenib (Figure 2), and the SERPING1 level was also detected in conditioned medium (Figure 2F). In addition, we successfully established SR HepG2 and Huh7 cells, and cell viability was not obviously influenced by sorafenib (Figure 3A,B). The lower SERPING1 levels in SR cells contribute to enhanced migratory capacity, which can be notably attenuated by SERPING1 stimulation (Figure 6). Collectively, our data highlight a novel regulatory mechanism of SERPING1, which might serve as a critical determinant of cancer progression and drug resistance in HCC. However, the detailed molecular mechanism and predictive role of SERPING1 in SR HCC progression need to be elucidated in more detail.

HCC treatment remains suboptimal, with targeted therapeutic agents often exhibiting poor efficacy and leading to acquired resistance in tumors following exposure [13]. To address this challenge, we conducted an analysis of sorafenib resistance‐related gene expression profiles using data from the Gene Expression Omnibus database (GSE94550) and Roessler Liver microarray datasets, integrating clinical information from the Oncomine database [14]. Our objective was to identify potential candidates associated with HCC sorafenib resistance and elucidate their correlations with clinical features. Through database analysis, we discovered that SERPING1 is significantly associated with overall survival, recurrence‐free survival, AFP level, metastasis risk signature score, and advanced pathological stage (Figure 1C–H). Additionally, SERPING1 was found to be upregulated by sorafenib treatment (Figure 2D–F). Previously, SERPING1 was demonstrated to be correlated with HCC development and associated with poor differentiation in HCC [15]. Vandsemb et al. reported that SERPING1 influences cell motility and plays a role in prostate cancer progression [16]. The present study revealed for the first time that SERPING1 is regulated by sorafenib, which in turn reduces HCC cell migration might through the upregulation of p‐ERK and increased activity of MMP‐2 and MMP‐9 stimulated with sorafenib. In the future, the mechanisms underlying the role of SERPING1 in sorafenib resistance and HCC progression still need to be elucidated extensively.

Previous studies by Chen et al. have demonstrated that sorafenib induces phosphorylation of ERK in various HCC cell lines, including Hep3B, JHH‐7, and HLF cells. Sorafenib, a RAF inhibitor, rapidly promotes RAF dimerization and subsequent ERK activation, which may contribute to therapeutic resistance. Chen et al. reported that the transactivation of ERK signaling and RAF dimer formation enhance HCC cell survival, inhibit apoptosis by downregulating Bim, and mitigate immunosuppression through the activation of PD‐L1 gene expression [17]. These findings suggest that while targeting cell proliferation pathways, compensatory activation of downstream signaling pathways could provide alternative survival mechanisms for HCC cells. Additionally, Chen et al. identified a correlation between elevated ERK phosphorylation levels and sorafenib‐induced necrosis in mouse liver cancer models. Increased ERK phosphorylation was positively associated with a reduction in the size of individual HCC nodules following sorafenib treatment [18]. Recently, Zhang et al. observed that sorafenib slightly induces ERK phosphorylation in the HCC cell line SMMC7721, potentially via the RAF–MEK signaling cascade, as evidenced by Western blot analysis [19]. Furthermore, we exposed HCC cells (Huh7 and J7) to 5 μM sorafenib for 24 h. Interestingly, we observed a decrease in p‐ERK levels following this treatment. This finding suggested a complex regulatory effect of sorafenib on p‐ERK. We speculate that short‐term sorafenib exposure may activate p‐ERK as part of a compensatory mechanism, which could support alternative survival pathways such as autophagy in HCC cells. In contrast, prolonged sorafenib treatment appears to induce substantial toxicity, thereby disrupting these compensatory mechanisms and leading to a reduction in p‐ERK levels.

Epithelial mesenchymal transition (EMT) is a dynamic process in which epithelial cells lose their cell polarity and cell–cell adhesion and convert into the mesenchymal type [20] Recently, accumulating evidence has indicated that EMT is involved in cancer cell metastasis and drug resistance in numerous cancers, in which numerous molecules, such as E‐cadherin, N‐cadherin, vimentin, twist 1/2, snail 1/2, and ZEB 1/2, could participate [21, 22, 23]. Coussens and colleagues have also shown that the tumor microenvironment is similar to the inflammatory response in wound repair, which influences turnover of the extracellular matrix (ECM) and tumor cell motility [24]. MMPs are involved in remodeling of the ECM and are necessary for numerous physiological events, such as wound healing and tumor metastasis [25, 26]. In the present study, we found that SERPING1 can reduce cell migration in Huh7 and J7 cells (Figure 4). Therefore, we suggest that EMT‐related molecules, including E‐cadherin, N‐cadherin, vimentin, twist 1/2, snail 1/2, and ECM‐associated factors, such as pro‐MMP‐2, active MMP‐2, pro‐MMP‐9, and active MMP‐9, might be involved in SERPING1‐regulated cell motility, which can be investigated extensively in the future. Additionally, SERPING1 is a protease inhibitor belonging to the serpin superfamily [3]. EMT is a dynamic process in which epithelial cells lose their cell polarity and cell–cell adhesion and convert into the mesenchymal type [20]. Therefore, EMT‐ or MMP‐related pathways, including MEK, p38, JNK, PI3K, Akt, mTOR, and ERK signaling [27], could be studied in more detail in sorafenib‐sensitive and SR cells.

Previously, Fornvik and colleagues demonstrated that the survival rate is increased in rats inoculated intracerebrally with glioma cells precoated with anti‐SERPING1 antibody [7]. Furthermore, the phenotypes of smaller tumors and increased survival appeared in a subcutaneous glioblastoma model with intratumoral treatment [28]. According to the evidence, we speculated that SERPING1 might play a role in cell proliferation, and this could be determined by SERPING1 overexpression and sorafenib stimulation in sorafenib‐sensitive and SR cells. Based on our findings, we suggest that SERPING1 is emerging as a key component for modulating cancer metastasis in SR HCC cells. Elucidation of the predictive role and molecular and cellular mechanisms of SERPING1 related to sorafenib resistance can provide additional opportunities to establish complementary therapies for HCC.

## Author Contributions

Formal analysis: C.‐C.H., C.‐I.W., C.‐J.L., T.‐C.H., and C.‐Y.C. Writing – original draft preparation: C.‐C.H., P.‐S.H., and C.‐Y.C. Writing – review and editing: I.‐H.L., C.‐W.C., Y.‐L.C., and C.‐Y.C. Performed the experiments: Y.‐H.W., Y.‐H.L., and P.‐S.H. Funding acquisition: C.‐C.H., T.‐C.H., and C.‐Y.C.

## Ethics Statement

The manuscript did not use human sample.

## Consent

The authors agree to publish.

## Conflicts of Interest

The authors declare no conflicts of interest.

## Supporting information


**Table S1** Prognostic significance of clinicopathologic indicators and SERPING1 for recurrence‐free survival in the Roessler liver array.

## Data Availability

It is already provided as part of the submitted article.
