# Supplementary material for: SERPING1 Reduces Cell Migration via ERK‐MMP2‐MMP‐9 Cascade in Sorafenib‐ Resistant Hepatocellular Carcinoma
Source: Environ Toxicol. 2024 Oct 30;40(2):318–27. doi: 10.1002/tox.24434 (PMC11726270; doi:10.1002/tox.24434)
Supplement: Supplementary file 1 — Table S1 Prognostic significance of clinicopathologic indicators and SERPING1 for recurrence‐free survival in the Roessler liver array. [file TOX-40-318-s001.docx]

**Supplementary Table 1.** **Prognostic significance of clinicopathologic indicators and SERPING1 for recurrence-free survival in the Roessler liver array.**

|  | RFS univariate | | | | RFS multivariate | | | |
| --- | --- | --- | --- | --- | --- | --- | --- | --- |
| Factor | Group | HR | 95% CI | P |  | HR | 95% CI | P |
| Age | <60/≥60 years | 0.952 | 0.628-1.443 | 0.817 |  |  |  |  |
| Sex | Female/Male | 2.359 | 1.238-4.493 | 0.009* |  | 2.293 | 1.203-4.370 | 0.023* |
| Cirrhosis | -/+ | 2.003 | 0.936-4.287 | 0.074 |  |  |  |  |
| Serum AFP | <300/≥300 ng/ml | 1.314 | 0.937-1.842 | 0.113 |  |  |  |  |
| Tumor size | <5/≥5 cm | 1.424 | 1.008-2.012 | 0.045* |  |  |  | NS |
| CLIP | 0-1/≥2 | 1.872 | 1.267-2.766 | 0.002* |  |  |  | NS |
| AJCC stage | I / ≥II | 1.968 | 1.386-2.794 | <0.001* |  | 1.906 | 1.342-2.706 | <0.001* |
| SERPING1 | Low/High | 0.681 | 0.486-0.954 | 0.026* |  |  |  | NS |

^*^P < 0.05. RFS, recurrence-free survival; CLIP, Cancer of the Liver Italian Program score; AJCC, American Joint Committee on Cancer 2017; AFP, alpha-fetoprotein.
